# Surgical Treatment of Necrotizing Pancreatitis

**DOI:** 10.3390/jcm15218380

**Published:** 2026-10-28

**Authors:** Apostolos Gaitanidis, Jay N. Collins

**Affiliations:** Department of Surgery, Eastern Virginia Medical School, Norfolk, VA 23507, USA

**Keywords:** pancreatitis, necrotizing, vard, necrosectomy, review, acute pancreatitis, pancreatic necrosis, infected pancreatic necrosis, step-up approach

## Abstract

Necrotizing pancreatitis is associated with significant morbidity and mortality, particularly in cases with infected necrosis. The management of these complex patients has radically shifted in the past 2 decades with major advances in the percutaneous, endoscopic and minimally invasive surgical techniques employed in the care of these patients. The step-up approach, incorporating percutaneous drainage and endoscopic transgastric necrosectomy, reserving surgical debridement for a small subset of patients, has significantly improved clinical outcomes. Delayed and minimally invasive approaches are now preferred to traditional open necrosectomy, with laparoscopic transgastric necrosectomy as an option. Transgastric necrosectomy has been established as a first-line treatment for debridement. Video-assisted retroperitoneal debridement (VARD) and sinus tract endoscopy (STE) are the most useful surgical approaches and are invaluable in cases where percutaneous drainage or endoscopic transluminal necrosectomy prove inadequate or incomplete. Open necrosectomy is reserved for rare cases with refractory infection, anatomical constraints, concurrent intra-abdominal pathology and lack of familiarity with minimally invasive approaches, but timely transfer to a regional center of excellence is encouraged. This review summarizes the evolution of contemporary surgical techniques, highlights the best evidence and delineates the role of each technique in a multidisciplinary framework.

## 1. Background on Necrotizing Pancreatitis

Acute pancreatitis is the most common gastrointestinal disease worldwide requiring hospital admission, with an annual incidence of 34 cases per 100,000 person-years among high-income countries [1]. Necrotizing pancreatitis develops in 10–30% of these patients and has a more complex course [2,3]. The multidisciplinary care for necrotizing pancreatitis has revolutionized in the past two decades due to the emergence of the step-up approach, dedicated endoscopic techniques and minimally invasive surgical techniques. Mortality is high in necrotizing pancreatitis, attributed to prolonged recovery times, debilitation related to the underlying disease process, multi-organ system involvement and the need for repeat interventions. Mortality rates range between 20 and 40% in pancreatitis where necrosis or organ failure has developed [1]. Sterile necrosis has a 10% mortality rate, while infected necrosis is associated with a 20–30% mortality rate [2,4,5]. With the introduction of endoscopic and minimally invasive interventions, mortality rates have significantly dropped in recent years [6]. Early deaths in acute pancreatitis are attributed to multi-system organ failure [7], and later deaths are typically attributed to the development of necrotic collections or infection of pancreatic necrosis [5].

Fluid collections in necrotizing pancreatitis are classified into four broad categories, based on two criteria: the presence of necrosis and timing relative to symptom onset. In this manner, an acute peripancreatic fluid collection (APFC) involves a sterile fluid collection within 4 weeks of symptom onset in the absence of necrosis. An acute necrotic collection (ANC) refers to a necrotic collection less than 4 weeks after symptom onset. A pancreatic pseudocyst is an organized, non-necrotic fluid collection occurring more than 4 weeks after symptom onset, and walled-off pancreatic necrosis (WOPN) is a necrotic collection occurring more than 4 weeks after symptom onset [1]. Many of these collections may be asymptomatic and not require treatment, but they can also develop secondary infection, or cause pseudoaneurysms of the splenic artery and its branches, portal vein thrombosis, gastric outlet obstruction and intestinal fistulas, among other problems. Given the rapidly evolving landscape in necrotizing pancreatitis, we aim to explore and better define the current role of surgical management of necrotizing pancreatitis. Although nonoperative, percutaneous, and endoscopic approaches are discussed to provide the necessary clinical context, the primary focus of this review is the contemporary role, indications, technical considerations, and outcomes of surgical interventions for necrotizing pancreatitis.

## 2. Management and Techniques

This manuscript was designed as a narrative review of the evolution and contemporary surgical management of necrotizing pancreatitis. Relevant literature was identified through searches of MEDLINE and Scopus through April 2026, using combinations of terms related to necrotizing pancreatitis, pancreatic necrosectomy, surgical debridement, minimally invasive surgery, endoscopic necrosectomy and the step-up approach. Given the narrative nature of this review, formal systematic screening and risk-of-bias assessment were not performed.

### 2.1. Open Necrosectomy and Historical Evolution

Open necrosectomy used to be commonplace prior to the shift towards the step-up approach and was the definitive treatment to pancreatic necrosis in the 1980s and 1990s. Surgeons were aggressive at performing surgical debridements, with common indications being signs of sepsis, organ failure, gastrointestinal bleeding, persistent paralytic ileus, or uncontrolled pain [8]. Surgery occurred as early as day 3 after the onset of acute pancreatitis, with organ failure deemed an indication for intervention. Nevertheless, the exact timing depended on several clinical factors [8]. Given those indications, many debridements were performed for sterile necrosis, something that would be exceedingly rare today [9]. Bradley et al. described that patients with sterile pancreatic necrosis may be treated without intervention, even when accompanied by organ failure, which started shifting the pendulum towards conservative management [10].

Open pancreatic debridements used to be quite aggressive. Several different techniques have been described, including necrosectomy with open packing, planned relaparotomies with repeated lavage, closed continuous lavage of the lesser sac and closed packing [11]. Necrosectomy with open packing was advocated by authors from Emory, and it involves open lavage and unroofing of the necrotic cavities, with the lesser sac then packed by non-adherent material and plans for re-exploration in 24–48 h for further debridement [12]. Planned relaparotomy with repeat lavage was advocated by surgeons at Mayo Clinic, and it involves open necrosectomy with extensive irrigation, temporary abdominal closure and subsequent return to the operating room at regular intervals, with eventual closure over suction drains [13]. Closed continuous lavage of the lesser sac and retroperitoneum involves the open exploration of the lesser sac, removal of all necrotic tissue and subsequent drain placement after suture closure of the gastrocolic ligament. This was advocated by authors from Ulm, Germany. Lavage through the drainage systems placed at the time of the operation is then performed postoperatively, and measurement of drain amylase or trypsin levels is used to guide cessation of the lavage [8]. Finally, closed packing refers to open necrosectomy, followed by the placement of stuffed Penrose drains and Jackson–Pratt drains [14]. This was advocated by authors at Massachusetts General Hospital. The Penrose drains are pulled back slowly to allow the cavity to close gradually [11]. Pancreatic resections were sometimes advocated in the 1980s, but with growing experience, they were abandoned in favor of parenchymal-sparing necrosectomies [8]. In centers with experience, mortality rates were reported to be less than 10% [8]. Given the morbidity of those procedures, it is not surprising that they have fallen out of favor. An infected pancreatic necrosis contains a large microbial count and high concentrations of pancreatic enzymes, both of which create very unfavorable conditions for wound healing. Arguably, the aggressive dissection of the retroperitoneal planes with open necrosectomy gets rid of the tissue that normally contains the infection in the retroperitoneal space and creates the opportunity for irritating pancreatic secretions and bacteria to spread further into the peritoneal cavity.

Management has now fundamentally shifted from decades ago when early, aggressive surgical intervention was advocated [15]. Gradually, evidence accumulated that “less is more” in the management of necrotizing pancreatitis [16]. One of the first milestones was the realization that sterile necroses did not require intervention. Aggressive surgical debridement was commonly advocated for necrotizing pancreatitis, even in the absence of infection. In 1991, Bradley et al. reported that 11 patients with sterile necrosis were successfully managed without surgical debridement with favorable outcomes, suggesting that conservative management may be superior to debridement even in the setting of organ failure [10]. This paradigm-shifting evidence demonstrated that sterile necrosis was best managed by observation, and additional studies further established this practice.

The second milestone came later in the decade, when it was demonstrated that even in the setting of infected necrosis, debridement is not an emergency and outcomes are better with later intervention. In 1997, Mier et al. randomized patients to early versus late debridement for infected pancreatic necrosis and found that mortality was 3.4 times higher with early compared to late debridement. While now regarded as standard practice, early source control was viewed as a cornerstone of management during that era. The next major milestone was the realization that many cases may be treated without debridement, as antibiotics and/or drainage may be all that is needed. In the late 2000s, it was reported that up to 62% of patients with necrotizing pancreatitis can be treated without debridement, as only 33% of patients required debridement, with the majority being treated with antibiotics and/or catheter drainage alone [17].

### 2.2. Pre-Intervention Management

There are several important determinations that need to be made when treating patients with necrotizing pancreatitis. The first step is to determine the presence of infection. The American Gastroenterological Association (AGA) guidelines recommend that infected necrosis should be suspected when cross-sectional imaging demonstrates gas in a peripancreatic fluid collection, or when clinical features, including persistent fever, worsening leukocytosis, persistent unwellness, or clinical deterioration, are present [2]. As necrotizing pancreatitis is often associated with a robust systemic inflammatory response, this determination on clinical grounds alone can be challenging. If infection is suspected, broad-spectrum antibiotics with pancreatic penetration should be initiated, often carbapenems or a combination of metronidazole and cephalosporins [2]. CT-guided fine-needle aspiration was once routinely performed for the diagnosis of infected necrosis but has fallen out of favor due to the high risk of false negatives and potential contamination of sterile collections [2]. To guide antibiotic selection in these patients, obtaining appropriate cultures after CT-guided percutaneous drainage is a useful adjunct to a therapeutic intervention. The use of prophylactic antibiotics to prevent infected necrosis or systemic complications has not shown any effectiveness and is not recommended [2].

The AGA [2] and ACG [18] guidelines recommend delaying intervention, preferably until approximately 4 weeks after disease onset, to allow maturation of necrotic collections, provided the patient remains clinically stable. The practice-changing PANTER trial was the one that truly shifted management towards a step-up approach, focusing on delaying intervention and avoiding aggressive debridement [19].

### 2.3. Step-Up Approach

The step-up approach has gained widespread acceptance for the treatment of infected necrosis. Contemporary use of this term refers broadly to a staged, individualized management strategy, ranging from initial conservative treatment with antibiotics to drainage and, when necessary, endoscopic or minimally invasive surgical debridement [2]. This does not represent a rigid sequence, as treatment selection depends on collection anatomy, clinical status, response to previous interventions and institutional expertise.

Recent findings suggest that a substantial number of patients can be treated with simply antibiotics or antibiotics and drainage alone, if patients do not improve with antibiotics alone. The POINTER trial assessed timing of intervention. One hundred and four patients were randomized to immediate drainage within 24 h versus postponed drainage until the stage of walled-off necrosis was reached. Although mortality did not differ between the two groups, immediate drainage was not superior to postponed drainage and delayed intervention resulted in fewer procedures required overall, as 39% of patients did not require any drainage at all [3]. This study reaffirms that a postponed drainage strategy is associated with fewer interventions and that an initial approach with just antibiotics is warranted upon initial diagnosis of infected necrosis (Table 1).

For patients that do not improve with antibiotics and catheter drainage alone, debridement can be performed, either through endoscopy or minimally invasive surgical approaches [2]. The PANTER trial [19] was pivotal in establishing the surgical step-up strategy, consisting of initial percutaneous or endoscopic drainage followed, when necessary, by video-assisted retroperitoneal debridement (VARD). This differs from subsequent endoscopic step-up strategies, in which transmural drainage is followed, if necessary, by direct endoscopic necrosectomy. Eighty-eight patients with infected pancreatic necrosis were randomized to open necrosectomy versus a step-up approach. The results showed a lower incidence of the combined primary outcome of major complications or death with the step-up approach compared to the traditional open approach. Morbidity was primarily related to new-onset organ failure, incisional hernias and pancreatic fistulas, all of which were more common in the open group [19]. Additional studies further defined the role of endoscopic debridement. The PENGUIN trial [20] showed a lower incidence of major complications or death with endoscopic transgastric necrosectomy compared to surgical necrosectomy, but it had a low sample size of 20 patients. The TENSION trial was conducted between 2011 and 2015 and randomized patients with infected pancreatic necrosis to an endoscopic or a surgical step-up approach. Although the endoscopic approach was not superior to the surgical approach with regards to the composite outcome of major complications or death, the rates of pancreatic fistula and hospital stay benefited the endoscopic group [21]. Another important trial was the MISER trial, a single-center study that randomized patients to an endoscopic transluminal approach or a minimally invasive surgical approach for necrotizing pancreatitis. The endoscopic approach was associated with fewer major complications and better quality-of-life scores at 3 months, further supporting endoscopy as the preferred option [22]. The rate of pancreatic fistulas was also lower in the endoscopic group [22]. Another large international, multi-center review of 1980 patients showed that both minimally invasive necrosectomy and endoscopic necrosectomy had lower risk of death compared to open necrosectomy without a direct head-to-head comparison [23]. The mortality benefit for endoscopic necrosectomy remained in the high-risk and very-high-risk groups [23]. These practice-changing studies have thoroughly changed the treatment landscape for necrotizing pancreatitis, and recent data suggests that the use of open necrosectomy has significantly declined in the United States [24]. Other authors are also pushing the envelope by examining robotic-assisted techniques in pancreatic debridement or cystogastrostomy with low perioperative morbidity and mortality [25]. Endoscopic ultrasound (EUS)-guided drainage using lumen-apposing metal stents (LAMSs) has facilitated transmural drainage and subsequent direct endoscopic necrosectomy. Mangiavillano et al. reported favorable technical and clinical outcomes with the novel Hot-Spaxus system across various EUS-guided interventions, including pancreatic fluid collection drainage [26].

**Table 1:** Summary of important randomized control trials in necrotizing pancreatitis.

Trial Name	Year of Publication	Inclusion Criteria	Main Outcomes
PANTER (van Santvoort et al.) [19]	2010	Adults with infected necrotizing pancreatitis requiring intervention. Step-up: *n* = 43 Open: *n* = 45	Step-up approach reduced major complications or death (40% [step-up] vs. 69% [open], *p* = 0.006). Mortality: 19% vs. 16%. New-onset organ failure: 12% vs. 42%. Incisional hernia: 7% vs. 24%. Pancreatic fistula: 28% vs. 38%
PENGUIN (Bakker et al.) [20]	2012	Patients with infected pancreatic necrosis suitable for either endoscopic or surgical necrosectomy. Endoscopic: *n* = 10 Surgical: *n* = 10	Endoscopic transgastric necrosectomy reduced inflammatory response (IL-6) and composite major complications or death compared to surgical necrosectomy (20% [endoscopic] vs. 80% [surgical], *p* = 0.03). Pancreatic fistula: 70% (surgical) vs. 10% (endoscopic, (*p* = 0.02), new-onset multiple organ failure: 50% (surgical) vs. 0 (endoscopic, *p* = 0.03).
TENSION (van Brunschot et al.) [21]	2018	Patients with infected pancreatic necrosis requiring intervention. Endoscopic: *n* = 51 Surgical: *n* = 47	No significant difference in death or major complications (endoscopic 43% vs. surgical 45% [NS]). Mortality 18% (endoscopic) vs. 13% (surgical), NS. Endoscopic approach had fewer pancreatic fistulas (5% vs. 32%) and shorter hospital stay (53 vs. 69 days).
MISER (Bang et al.) [22]	2019	Patients with walled-off necrosis requiring intervention. Endoscopic: *n* = 34 Surgical: *n* = 32	Endoscopic therapy reduced composite outcome of major complications or death (11.8% vs. 40.6%, *p* = 0.007). Pancreatic fistulas lower with endoscopic (0 vs. 28.1%). Lower healthcare costs ($75,830 endoscopic vs. $117,492 surgery, *p* = 0.039), improved QoL at 3 months (*p* = 0.007), mortality similar.
POINTER (Boxhoorn et al.) [3]	2021	Patients with infected pancreatic necrosis eligible for drainage. Immediate: *n* = 55 Postponed: *n* = 49	Immediate vs. postponed drainage: no benefit to early drainage in composite outcome of complications or death (Comprehensive Complication Index score: 57 vs. 58, Mortality within 6 months: 13% vs. 10%). Delayed strategy reduced interventions, with many patients avoiding drainage entirely (100% vs. 61%).

### 2.4. Video-Assisted Retroperitoneal Debridement (VARD)

The success of the VARD technique has shifted the pendulum for patients with necrotizing pancreatitis. Initial reports of retroperitoneal necrosectomy via large incisions was made in the late 1980s [27], but it was not until the early 2000s when the first minimally invasive approaches to retroperitoneal debridement were described in the Netherlands [28]. The improvements in patient outcomes quickly led to the PANTER trial that established the current paradigm of care [19].

**Figure 1:**
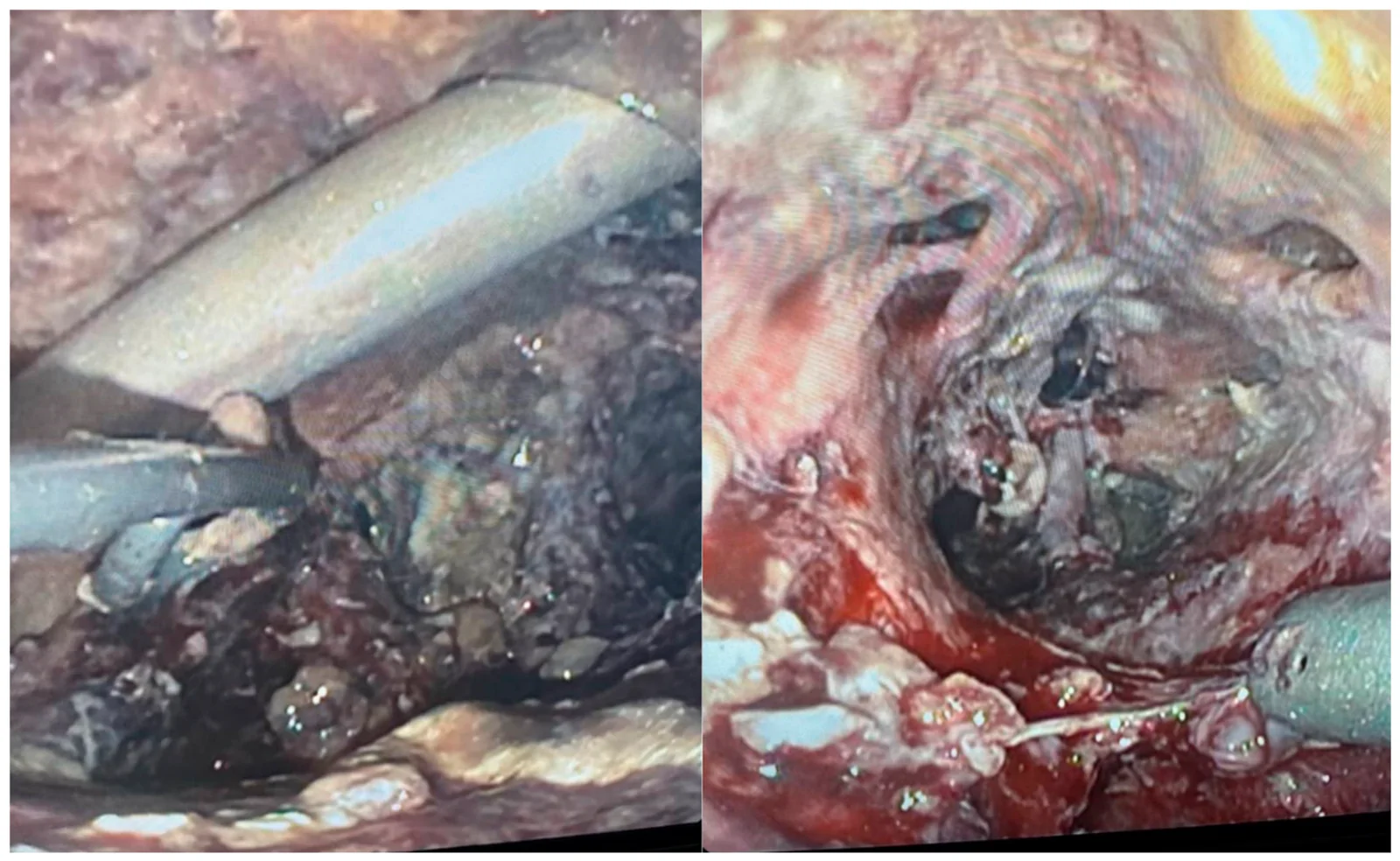
Intraoperative image obtained during video-assisted retroperitoneal debridement in a patient with extensive necrotizing pancreatitis. On the (**left**), the necrotic tissue is seen prior to debridement. On the (**right**), debridement is being performed. It is important to stop when oozing is encountered, as it implies healthy viable tissue.

The technique involves the initial placement of percutaneous drains via interventional radiology (IR) to the retroperitoneum, typically on the left side. One or two such IR drains may be placed in order to provide additional access points to the collection. Drains need to be upsized one to two times before the tract can be used for VARD [29]. Access to the collection is obtained through the drain tract. The incision is extended over the drain site and the tract is upsized. A laparoscope is then introduced into the collection. Inspection can be facilitated using CO_2_ gas insufflation. After insufflation, an additional trocar may be placed to assist with debridement and irrigation. Secondary laparoscopic instruments can be used to debride necrotic material, suction and irrigate. The focus of debridement is on loosely adherent pieces of necrotic material and avoiding injuring retroperitoneal blood vessels due to the difficulty in obtaining hemostasis [28]. Another useful adjunct for visualization of the necrotic collection is advancement of a gastroscope through the transgastric stent placed during endoscopic necrosectomy, permitting direct endoscopic inspection of the cavity. After the bulk of the necrotic material is evacuated, the cavity is thoroughly irrigated. The goal of the procedure is not to perform a complete necrosectomy, and additional procedures may be needed for complete debridement (Figure 1). At the end of debridement, two large bore drains are placed in the cavity. These drains can be used for postoperative lavage if desired. Suction drains may or may not be used, but most importantly, their caliber should be adequate to allow drainage and/or irrigation. Postoperative imaging can be obtained to evaluate the extent of debridement performed and whether additional procedures are necessary [28].

The perioperative physiological stress is much less compared to open necrosectomy, and this has been confirmed by randomized controlled trials. Retrospective studies have also shown a lower mortality rate with such an approach [6]. Compared to open necrosectomy, peritoneal contamination is avoided, and this reduces physiological insults. VARD has several advantages compared to sinus-tract endoscopy, which is described later in this paper. Because the incision is bigger, larger pieces of necrotic tissue can be removed. No fluoroscopy is needed. Fewer procedures are typically needed, as the technique is more versatile than sinus-tract endoscopy. The incision, however, is larger, so wound complications and pancreaticocutaneous fistulas are more likely [28]. Compared to endoscopic transgastric necrosectomy, VARD usually requires fewer sessions to achieve complete debridement. In experienced hands, complication rates are low, and only one procedure is required to fully drain the necrotic collections [29]. Obesity can make the procedure more challenging, as the tract to the retroperitoneum is longer and the amount of retroperitoneal fat is greater, but an experienced operator should be able to complete the procedure without conversion to an open necrosectomy [29]. Medial collections with extension to the mesenteric root are particularly challenging for VARD, as the distance from the entry point is longer and there are important structures nearby. Often, these are close to the stomach and can be managed with transgastric drainage and avoidance of open necrosectomy [29]. Hybrid procedures combining both endoscopic drainage and VARD have also been described, termed dual-modality drainage [30], and they can be useful in cases of necrotizing pancreatitis with intestinal fistulas [31].

Experience with this technique has spread globally [23] but tends to coalesce in regional centers of excellence given the rarity and learning curve associated with it. Van Brunschot et al. [23] performed a large retrospective multicenter analysis of patients undergoing surgical necrosectomy and found lower mortality of minimally invasive approaches like VARD compared to open necrosectomy. A subset of very-high-risk patients also had a lower risk of mortality with this approach, proving that the lower physiological stress is translated to improved clinical outcomes. Luckhurst et al. performed a large single-institution study at Massachusetts General Hospital and further showed that 91 patients undergoing a minimally invasive step-up approach had lower mortality compared to open surgery. A trend for higher risk of bleeding complications was noted, but these results did not lead to worse survival [6]. Gomatos et al. from Liverpool showed that a minimally invasive retroperitoneal necrosectomy had better survival than the open approach [32]. The same institution had previously reported lower rates of organ failure with this technique [33]. The most important cohorts from patients undergoing minimally invasive surgical debridement are shown in Table 2. Although randomized trials support the benefits of minimally invasive step-up strategies, much of the evidence comparing individual surgical techniques derives from retrospective observational studies, and their results should be interpreted with caution.

**Table 2:** Outcomes from the largest reported cohorts of minimally invasive surgical management of necrotizing pancreatitis.

Study	Publication Year	Sample Size in Surgical Group	Type of Surgery	Key Findings
van Brunschot et al. [23] Multicenter, International	2018	467	Minimally invasive surgical necrosectomy	Lower mortality risk than open necrosectomy (OR 0.53), including in very-high-risk patients (OR 0.70).
Luckhurst et al. [6] Boston, MA, USA	2020	91	Minimally invasive step-up approach, including 9% PD, 8% VARD, 15% STE, 32% ETN, 27% combination	Lower mortality with step-up approach compared to open (2% vs. 10%, *p* = 0.03). Lower rate of organ failure (30% vs. 45%, *p* = 0.029), higher bleeding rate (19% vs. 9%, *p* = 0.064).
Gomatos et al. [32] Liverpool, UK	2015	274	Minimal access retroperitoneal pancreatic necrosectomy	Mortality rate lower with minimally invasive (15.3%) vs. open (23.3%) approach (*p* = 0.064).
Raraty et al. [33] Liverpool, UK	2010	137	Minimal access retroperitoneal pancreatic necrosectomy	Organ failure lower with minimally invasive (31%) vs. open (56%) approach. Mortality also lower (19% vs. 38%, *p* = 0.009).
Rasch et al. [34] Multicenter, Germany	2016	190	Minimally invasive step-up approach, including PD, ETN, VARD	Lower rate of severe complications (44.7% vs. 73.3%), mortality (10.5% vs. 33.3%) and diabetes (4.7% vs. 33.3%).

PD: percutaneous drainage, VARD: video-assisted retroperitoneal debridement, STE: sinus-tract endoscopy, ETN: endoscopic transgastric necrosectomy.

### 2.5. Sinus-Tract Endoscopy

Sinus-tract endoscopy (STE) is one of the main minimally invasive surgical techniques used in the management of necrotizing pancreatitis. The technique was originally described by Carter et al. in 2000 [35], but its importance has been amplified given the shift in pancreatitis management with the advent of the step-up approach. The main difference compared to VARD is that it uses a dilational approach to gain entry into the collection, compared to a cutdown approach with VARD.

Access to the retroperitoneum is commonly obtained through a drainage catheter placed in a fluid collection by IR. The first step is to place a 0.035” super stiff wire through the existing catheter tract and confirm its position at the tip of the drain using fluoroscopy. The tract is then dilated to 30 Fr with a balloon and a plastic sheath is placed. Once access to the necrotic cavity is obtained, the nephroscope is inserted and connected to irrigation. Once the right position is confirmed, the wire is removed. A laparoscopic grasper can then be inserted through the scope and piecemeal removal of solid material is then performed. The nephroscope allows intermittent irrigation and suction, and the working channel can be used for instrumentation. Haptic feedback is crucial, as loose debris is necrotic, while firm tissue should be left intact. Once the initial debris is cleared, it becomes easier to obtain visualization. Visualization can be challenging even with small amounts of oozing, so the operator needs to be ready for that. After the debridement is complete, a new drain is placed in the cavity and its position is confirmed with fluoroscopy [35,36].

The main advantage of STE is that it is not as bound by the same anatomic constraints as VARD [36]. STE can access most collections that are accessible to IR via the drain catheter. There are several examples of collections that might not be amenable to VARD. Necrotic collections that are only accessible via a transperitoneal route are easily accessible with STE but are not amenable to VARD. Similarly, collections where the best access route is intercostal might not be amenable to VARD, as the working space is too small for multiple working ports. Additionally, if the only available window to the necrotic collection is so narrow that VARD may risk injury to vital structures, STE might be the only option. STE has fewer wound complications compared to VARD, as it only uses a single incision.

The main drawback of the technique is the lack of familiarity with the rigid nephroscope and the learning curve to become facile at it. Additional procedures may also be necessary to obtain control of large collections. Bleeding can be challenging to control, so aggressive debridement of the cavitary walls should be avoided, opting instead for a repeat procedure [36]. The main risk associated with the procedure is the creation of a pancreaticocutaneous fistula, which can be managed conservatively in most cases. Aggressive debridement is also not necessary, as a lot of this material will separate with lavage and be removed after a repeat procedure [35]. The time required for STE is low, with an average of 45 min [37]. Between one and four sessions may be required for completion of the debridement. Lavage in between sessions can assist future sessions [37]. Outcomes are favorable, with mortality reported between 0 and 14.3% among different studies and typically due to the additional use of open necrosectomy in older cohorts [35,37,38]. The optimal interval between sessions and the endpoints during each session need to be better refined to optimize the yield of each session.

### 2.6. Indications and Timing of Surgery

In this ever-changing landscape of treatment paradigms, the role of surgery is continuously evolving. Interventions in necrotizing pancreatitis are mainly limited to the presence of infected necrosis [15], but, as stated previously, the diagnosis of infected necrosis may be challenging in the absence of pathognomonic radiologic findings. Delays in diagnosis and treatment can lead to septic shock and death [39]. In sterile necrosis, clinical judgment should guide the need for intervention. Unremitting abdominal pain, gastric outlet or biliary obstruction are among the most important indications for intervention in sterile necrosis [19,39]. These indications are supported primarily by observational evidence and expert consensus, in contrast to the stronger evidence supporting intervention in infected necrosis. Disconnected duct syndrome is another very late complication of necrotizing pancreatitis that will require surgical intervention, either as a Roux-en-Y pancreaticojejunostomy (or pancreaticogastrostomy) or distal pancreatectomy [2,18,40,41].

**Figure 2:**
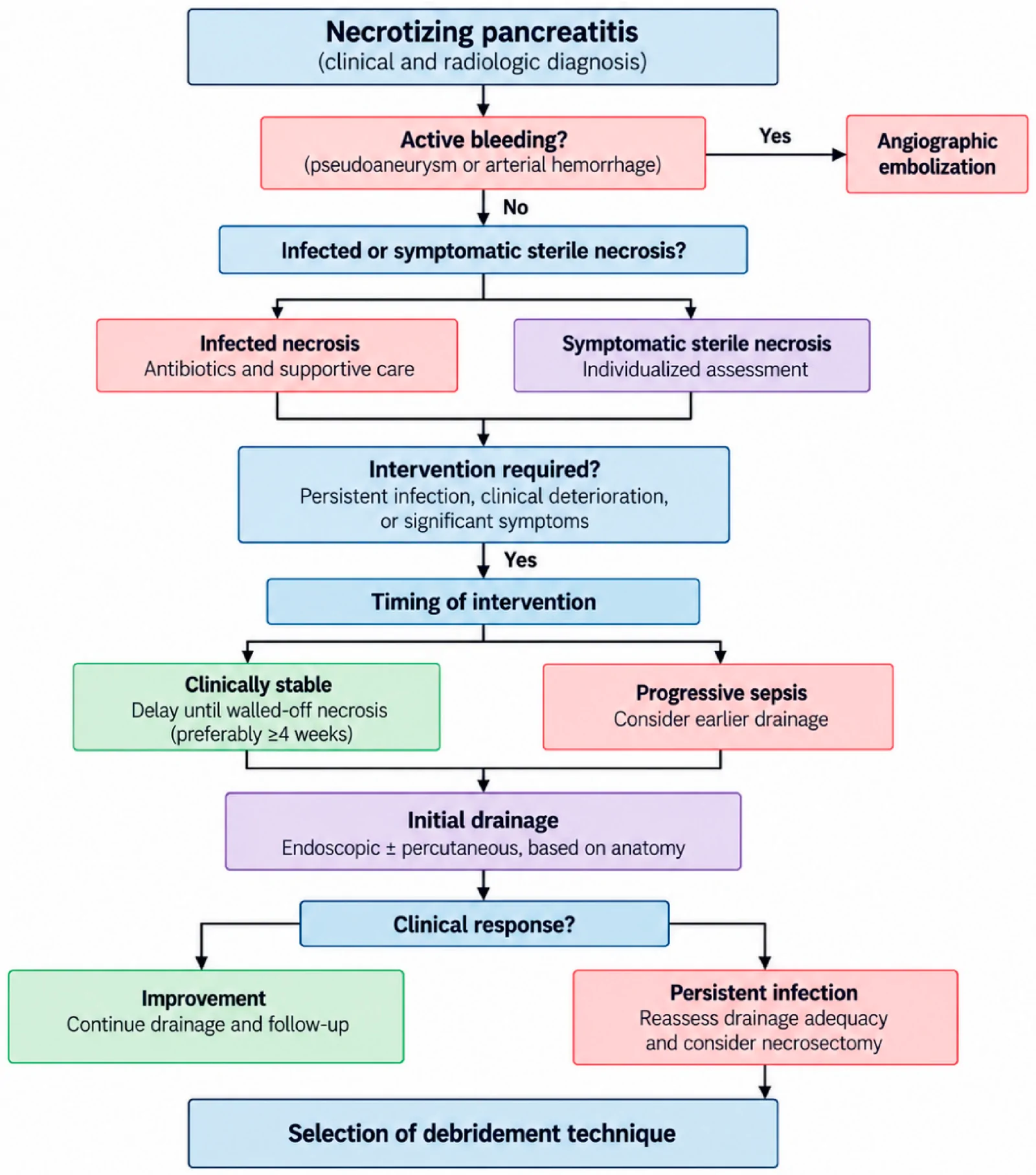
Necrotizing pancreatitis: from diagnosis to debridement.

As stated previously, the need for surgical debridement is dictated by the response to conservative management and percutaneous drainage, and it can be postponed for several weeks from disease onset. Urgent debridement is very rarely indicated, but urgent source control may be required, frequently through percutaneous or endoscopic drainage combined with appropriate antibiotics. In stable patients, intervention should preferably be delayed until collections mature, whereas progressive sepsis may necessitate earlier drainage. Persistent infection despite appropriate antibiotics and adequate percutaneous or endoscopic drainage should prompt consideration of necrosectomy. While surgery may rarely be required for other problems during the early phase of acute pancreatitis (e.g., bowel infarction or abdominal compartment syndrome), aggressive debridement during that time period is usually more likely to hurt than to help. However, in the era of the step-up approach, it is important not to forget that there are instances when deteriorating clinical condition necessitates aggressive treatment to prevent intra-abdominal sepsis and septic shock. Clinicians should have a high threshold of suspicion that patients may require surgical debridement and should actively optimize them in preparation for that. Given the growing comfort with the step-up approach, it is easy to dismiss signs of inadequate drainage or sepsis and fail to intervene surgically when necessary (Figure 2).

Hemorrhagic complications represent another important consideration in the management of necrotizing pancreatitis. Vascular erosion and pseudoaneurysm formation, most commonly involving the splenic or gastroduodenal arteries, can result in life-threatening hemorrhage. In patients with suspected arterial bleeding, multiphasic contrast-enhanced CT is the preferred initial diagnostic modality, followed by angiography when indicated. Importantly, the presence of an untreated pseudoaneurysm may influence the timing of drainage or necrosectomy, as manipulation or decompression of necrotic collections may precipitate hemorrhage. When feasible, vascular complications should therefore be addressed before proceeding with endoscopic or surgical debridement. Unlike uncomplicated necrotizing pancreatitis, active hemorrhage may necessitate urgent intervention regardless of the maturity of the necrotic collection.

### 2.7. Choice of Procedure

**Figure 3:**
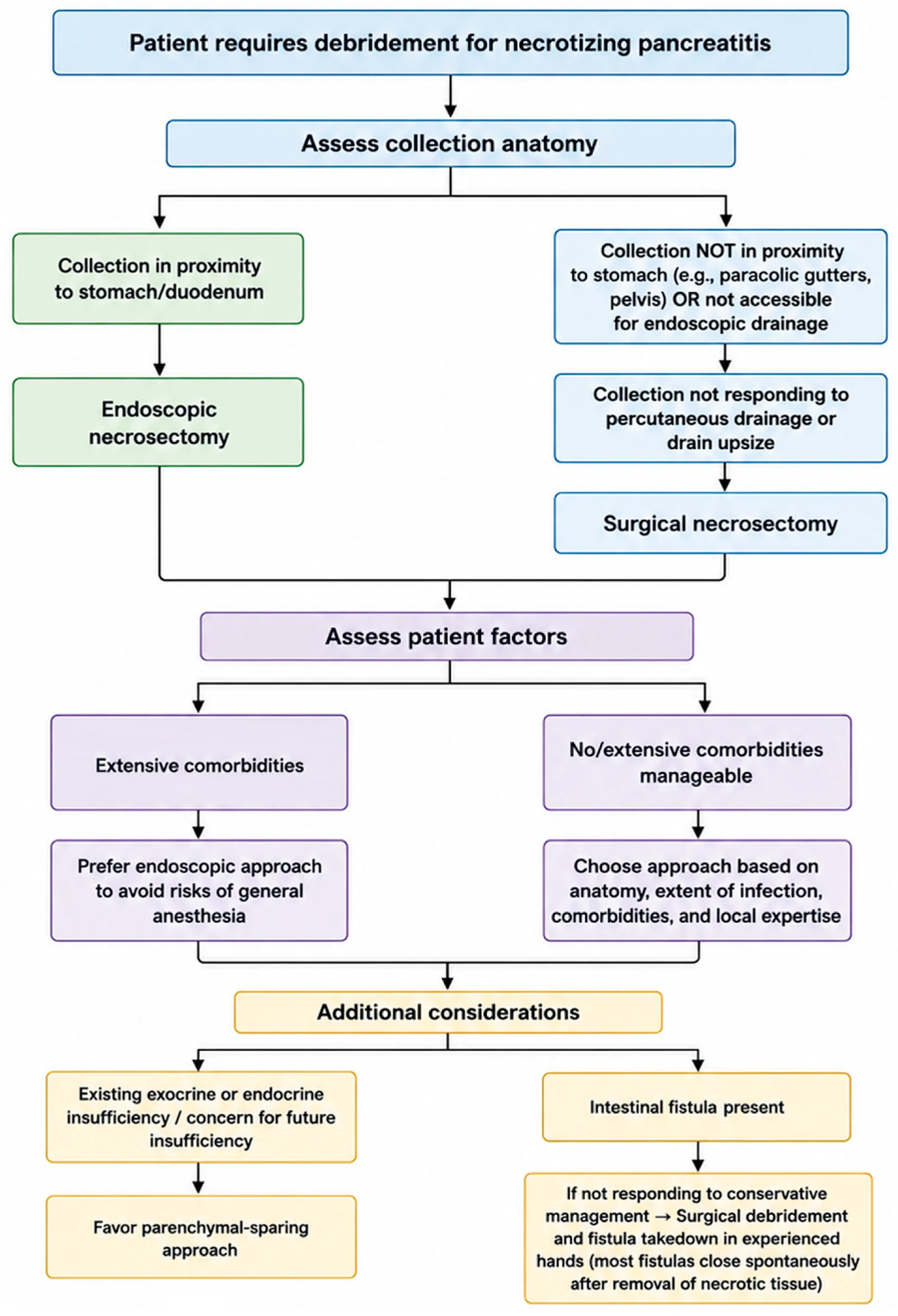
Algorithm showing section of appropriate debridement procedure.

The choice of specific debridement techniques depends on the patient’s clinical status, specific anatomical details, extent of intra-abdominal infection, comorbidities and regional availability. Anatomical details dictate the choice of appropriate techniques. Collections that are not responding to percutaneous drainage or drain upsize and are not in proximity to the stomach for endoscopic drainage should be treated with surgical necrosectomy. Necrotic retroperitoneal collections posterior to the ascending and descending colon, as well as the pelvis, are excellent candidates for surgical drainage given the large distance from the stomach to perform endoscopic transgastric necrosectomy (Figure 3). Patients with intestinal fistulas not responding to conservative management should also be offered surgical debridement and fistula takedown, but only in experienced hands, and most intestinal fistulas will close spontaneously after removal of necrotic tissue. The degree of exocrine and endocrine insufficiency can help dictate the need for a parenchymal-sparing approach. Patients with extensive comorbidities, such as liver cirrhosis or cardiopulmonary disease, may need to be managed endoscopically to avoid the risks of general anesthesia [2,18,40].

The indications of open necrosectomy in the current era are still debated. There are two factors to determine the need for open necrosectomy, namely inadequate source control despite less invasive procedures and lack of experience with minimally invasive approaches. In experienced hands, even the most complex necrotic collections may be managed with a combination of VARD, STE or transgastric necrosectomy. In less experienced centers, open necrosectomy is indicated in patients with infected necrosis and ongoing sepsis when less invasive procedures have failed. It is important to remember that while a minimally invasive procedure is preferred, in the absence of relevant experience and continued clinical decompensation, either a transfer to an institution with relevant experience or open necrosectomy is needed. Other rare indications include the need for additional intra-abdominal surgery, such as takedown of an intestinal fistula, abdominal compartment syndrome or disconnected duct syndrome. Given the morbidity of open necrosectomy and lack of experience in recent decades, priority should be given to timely transfer of these patients to an experienced referral center for further treatment. Formal training pathways should also be encouraged to create regional centers of excellence for this complicated disease process.

Recent guidelines, including those published by the World Society of Emergency Surgery (WSES) in 2019 [41], American Gastroenterological Association (AGA) in 2020 [2], American College of Gastroenterology (ACG) in 2024 [40] and International Association of Pancreatology (IAP) in 2025 [18], demonstrate substantial agreement regarding the management of necrotizing pancreatitis (Table 3). All discourage routine prophylactic antibiotic administration, support antibiotic treatment for infected necrosis, and favor delayed interventions and minimally invasive step-up approaches when able. Important differences exist regarding the timing of intervention and selection of drainage modalities. While WSES specifically recommends postponing surgical intervention beyond four weeks, the AGA advises against debridement during the first two weeks and the ACG recommends delaying any intervention in clinically stable patients for approximately four weeks. The 2025 IAP guidelines similarly favor delayed intervention until collections become walled off, generally after 3–4 weeks, but explicitly support earlier endoscopic or percutaneous drainage when conservative management fails and partial encapsulation is present.

Differences are more pronounced regarding the selection of drainage and debridement techniques. WSES recommends percutaneous drainage as the initial intervention for infected pancreatic necrosis, whereas the AGA and IAP recognize both percutaneous and endoscopic drainage as appropriate first-line approaches, with selection influenced by collection anatomy and institutional expertise. Regarding endoscopic drainage, the AGA generally favors lumen-apposing metal stents (LAMSs) over plastic stents, while the IAP recommends LAMSs particularly for infected walled-off necrosis or collections containing more than 30% necrotic tissue. The 2025 IAP guidelines also provide more detailed recommendations for escalation to necrosectomy, emphasizing persistent infection despite adequate drainage and antibiotic therapy and favoring retroperitoneal or endoscopic necrosectomy according to the established drainage route. Although all guidelines support minimally invasive approaches over conventional open necrosectomy when feasible, the IAP additionally recognizes single-stage surgical transgastric necrosectomy for collections predominantly localized to the lesser sac. Differences also exist regarding disconnected pancreatic duct syndrome, with the AGA recommending distal pancreatectomy in suitable patients with a disconnected left pancreatic remnant, whereas the IAP favors endoscopic management for symptomatic patients following unsuccessful conservative treatment. These recommendations reflect differences in procedural preferences and the specificity of clinical guidance rather than fundamental disagreement regarding the principles of necrotizing pancreatitis management. Furthermore, the WSES lists bowel fistulas extending into peripancreatic collections among the indications for surgical intervention, while the IAP distinguishes upper gastrointestinal fistulas, which generally resolve spontaneously, from colonic fistulas, which may require surgery in the setting of persistent infection, peritonitis, or feculent drainage.

## 3. Unresolved Questions and Future Directions

Despite significant advances in the management of necrotizing pancreatitis, several important questions remain unanswered. The optimal sequencing of endoscopic and surgical interventions, criteria for escalation following inadequate drainage and management of extensive or multifocal necrosis require further investigation. Complex anatomical considerations, including disconnected pancreatic duct syndrome, vascular complications and intestinal fistulas, may necessitate individualized or combined approaches. Furthermore, comparative evidence for individual minimally invasive surgical techniques remains limited, with most studies relying on observational data. Future research should focus on defining patient selection criteria and optimizing the timing and sequencing of interventions.

## 4. Conclusions

The management of necrotizing pancreatitis has changed dramatically in the past 2 decades. The paradigm shifted from early open surgical debridement to a nuanced, multidisciplinary care of complex patients in regional centers of excellence with minimally invasive approaches. Surgical management is continuously evolving. VARD and STE are useful modalities, offering reduced physiological stress and improved clinical outcomes compared to open necrosectomy. In the current era, endoscopic drainage is often preferred, due to the lack of pancreaticocutaneous fistula creation, but necrotic retroperitoneal collections posterior to the ascending and descending colon, as well as the pelvis, may necessitate surgical debridement if percutaneous drainage fails. Open necrosectomy is infrequently indicated, as many appropriately selected collections can be managed through minimally invasive or combined approaches. Such an approach may be necessary when other strategies are inadequate or unavailable. Nevertheless, timely transfer of these complex patients to experienced centers with multidisciplinary care is crucial to optimize clinical outcomes.

## Figures and Tables

**Table 3:** Comparison of international guideline recommendations for the management of necrotizing pancreatitis.

Domain	WSES 2019	AGA 2020	ACG 2024	IAP 2025
Management of sterile necrosis	Most patients with sterile necrosis can be managed conservatively (guideline discussion).	Intervention may be necessary for persistent symptoms or complications of sterile necrosis (BPA5).	Antibiotics should not be administered for sterile necrosis (KC15).	Conservative management is recommended during the early phase (R30; strong/low).
Prophylactic antibiotics	Routine prophylactic antibiotics are not recommended (1A).	Prophylactic antibiotics to prevent infection of sterile necrosis are not recommended (BPA2).	Suggests against prophylactic antibiotics in severe AP (R8; conditional/very low).	Prophylactic antibiotics are not recommended to prevent infectious complications (R19; strong/high).
Antibiotics for infected necrosis	Antibiotics are recommended for infected necrosis (2A). Use agents penetrating pancreatic necrosis with broad aerobic/anaerobic Gram-positive and Gram-negative coverage (1B).	Broad-spectrum IV antibiotics penetrating pancreatic necrosis are recommended for proven or strongly suspected infection (BPA2–3).	Antibiotics penetrating necrosis may allow intervention to be delayed beyond 4 weeks or drainage to be avoided in selected patients (KC15–16).	Initial conservative-first management with broad-spectrum IV antibiotics and supportive care is recommended (R17, R32–33).
Antifungal treatment	Routine prophylactic antifungals are not recommended (1B).	Routine antifungal administration is not recommended (BPA3).	Routine antifungal administration is unnecessary (KC17).	Prophylactic and empirical antifungal administration is not recommended (R34; conditional/low).
Failure of conservative management	Clinical deterioration with suspected infected necrosis warrants drainage (1C).	Persistent symptoms or complications may require drainage/debridement (BPA5).	—	Persistent fever/leukocytosis or deterioration with sepsis despite appropriate antibiotics and supportive therapy defines failure of conservative treatment (GPS35).
Indications for intervention	Deteriorating infected necrosis; after 4 weeks, persistent organ failure, gastrointestinal/biliary obstruction, disconnected duct syndrome, or symptomatic/enlarging collections; persistent pain after 8 weeks (1C).	Infected necrosis or symptomatic/complicated sterile necrosis, including obstruction, nutritional failure, fistulas, recurrent pancreatitis, or persistent SIRS (BPA5).	—	Deteriorating infected necrosis, prolonged symptomatic sterile WON, pancreatic hemorrhage, bowel ischemia, or fistulas (R36; strong/low).
Timing of intervention	Postponing surgical intervention beyond 4 weeks is associated with lower mortality (2B).	Avoid debridement during the first 2 weeks; optimally delay until approximately 4 weeks (BPA6).	Delay surgical, radiological, or endoscopic intervention in stable patients, preferably for 4 weeks (KC23).	Delay intervention until collections become walled off, generally after 3–4 weeks (R37; strong/low).
Early intervention	Percutaneous or endoscopic drainage is indicated when patients deteriorate with suspected infected necrosis (1C).	Percutaneous drainage may be considered within the first 2 weeks for infected/symptomatic collections or patients too ill for other interventions (BPA8).	—	Earlier drainage (<4 weeks) may be appropriate after failure of conservative management when collections are at least partially encapsulated (R38; conditional/low).
Initial drainage modality	Percutaneous drainage is recommended as first-line treatment for infected necrosis (1A).	Percutaneous or endoscopic transmural drainage are appropriate first-line approaches for WON; endoscopic drainage may be preferred when feasible (BPA7).	—	Either percutaneous or endoscopic transmural drainage is recommended according to anatomy and expertise (R41; strong/high). Endoscopic drainage is preferred when both approaches are feasible (guideline discussion).
Extensive or anatomically complex collections	—	Adjunct percutaneous drainage should be strongly considered for WON extending into the paracolic gutters or pelvis, or for residual necrosis after other interventions (BPA8).	—	Combined endoscopic, percutaneous, and/or retroperitoneal approaches may be necessary for extensive or multicompartment collections (discussion accompanying R41).
Endoscopic stent selection	—	LAMSs appear to be superior to plastic stents for transmural drainage of necrosis (BPA9).	—	Multiple plastic stents or LAMSs may be used; LAMSs are favored for infected WON or collections containing >30% necrosis (R42; strong/high).
Step-up approach	Percutaneous drainage is recommended first-line and may resolve infection without surgery in approximately 25–60% of patients (1A).	Initial percutaneous or endoscopic drainage, followed when necessary by direct endoscopic necrosectomy and surgical debridement, is reasonable; the sequence may vary according to expertise (BPA15).	—	Antibiotics followed by percutaneous/endoscopic drainage and, when necessary, minimally invasive retroperitoneal or endoscopic necrosectomy are recommended (R40; strong/high).
Indications for necrosectomy	Surgical escalation is considered when percutaneous or endoscopic treatment fails to improve the patient’s condition (1C).	Direct endoscopic necrosectomy is reserved principally for inadequate response to transmural drainage; it may also be used for extensive infected necrosis at expert centers (BPA10).	—	Necrosectomy is indicated for persistent fever/sepsis despite adequate antibiotics and drainage, or failure of antibiotics when drainage is infeasible (R43; strong/high).
Minimally invasive necrosectomy	Endoscopic necrosectomy and VARD are associated with less new-onset organ failure but may require more interventions (1B).	Minimally invasive approaches are preferred over conventional open necrosectomy because of lower morbidity (BPA11–12).	Minimally invasive methods are preferred in stable patients with symptomatic necrosis (KC22).	Minimally invasive approaches are preferred. Retroperitoneal necrosectomy is recommended through an established retroperitoneal drainage tract, and peroral endoscopic necrosectomy following transmural drainage (R44i–iii; strong/high).
Surgical transgastric necrosectomy	Single-stage surgical transgastric necrosectomy is an option for selected patients with WON or disconnected pancreatic duct (2C).	Laparoscopic and open transgastric debridement are feasible techniques (BPA12).	—	Single-stage laparoscopic or open transgastric necrosectomy is an effective option for WON predominantly localized to the lesser sac (R44iv; strong/high).
Open necrosectomy	Insufficient evidence establishes a mortality advantage of open, minimally invasive, or endoscopic approaches over one another (1B).	Open debridement remains appropriate when less invasive approaches are unsuitable (BPA13).	Minimally invasive approaches are preferred in stable symptomatic patients (KC22).	Open debridement is rarely indicated and should be reserved for selected predominantly solid collections and associated intra-abdominal complications (R44v; strong/high).
Complications requiring surgery	Surgical indications include refractory abdominal compartment syndrome, hemorrhage after failed endovascular treatment, bowel ischemia, necrotizing cholecystitis, and bowel fistula extending into a peripancreatic collection (1C).	Complications including obstruction and fistulas may warrant intervention (BPA5).	—	Hemorrhage, bowel ischemia, and fistulas may require intervention, with the approach determined by the complication (R36, R54).
Bowel fistulas	Bowel fistula extending into a peripancreatic collection is an indication for surgical intervention (1C).	Fistulas may necessitate intervention in complicated necrosis (BPA5).	—	Upper gastrointestinal fistulas generally resolve without intervention. Colonic fistulas may require surgery for persistent/worsening infection, peritonitis, or feculent drainage (R54; conditional/low).
Disconnected pancreatic duct syndrome	DPDS is included among indications for intervention; surgical transgastric necrosectomy may be considered in selected patients (1C, 2C).	Distal pancreatectomy is recommended for suitable patients with a disconnected left pancreatic remnant following mid-body necrosis; evidence for long-term transenteric stenting is insufficient (BPA14).	—	Intervention is indicated for persistent high-output pancreatic fistula, symptomatic recurrent collections, or recurrent upstream pancreatitis (R95). Endoscopic management is preferred after failure of conservative treatment (R96; conditional/low).
Multidisciplinary management and referral	Surgical treatment should be individualized by a multidisciplinary team according to local expertise (2C).	Multidisciplinary management is recommended, with consideration of tertiary referral when expertise is limited (BPA1).	—	Transfer to a high-volume center is recommended when local expertise is insufficient for patients with organ failure or infected necrosis (GPS6). Procedural selection should consider anatomy and expertise (R41).

ACG, American College of Gastroenterology; AGA, American Gastroenterological Association; AP, acute pancreatitis; BPA, Best Practice Advice; CRP, C-reactive protein; DPDS, disconnected pancreatic duct syndrome; FNA, fine-needle aspiration; GPS, Good Practice Statement; IAP, International Association of Pancreatology; KC, Key Concept; LAMS, lumen-apposing metal stent; R, Recommendation; SIRS, systemic inflammatory response syndrome; VARD, video-assisted retroperitoneal debridement; WON, walled-off necrosis; WSES, World Society of Emergency Surgery.

## Data Availability

No new data were created or analyzed in this study. Data sharing is not applicable to this article.
